# Attitudes to E-Cigarettes and Cessation Support for Pregnant Women from English Stop Smoking Services: A Mixed Methods Study

**DOI:** 10.3390/ijerph16010110

**Published:** 2019-01-03

**Authors:** Sue Cooper, Sophie Orton, Katarzyna A. Campbell, Michael Ussher, Naomi Coleman-Haynes, Rachel Whitemore, Anne Dickinson, Andy McEwen, Sarah Lewis, Felix Naughton, Katharine Bowker, Lesley Sinclair, Linda Bauld, Tim Coleman

**Affiliations:** 1 Division of Primary Care, University of Nottingham, Tower Building, University Park, Nottingham NG7 2RD, UK; sophie.orton@nottingham.ac.uk (S.O.); kasia.campbell@nottingham.ac.uk (K.A.C.); naomi_colemanhaynes@aol.co.uk (N.C.-H.); rachel.whitemore@nottingham.ac.uk (R.W.); anne.dickinson@nottingham.ac.uk (A.D.); katharine.bowker@nottingham.ac.uk (K.B.); tim.coleman@nottingham.ac.uk (T.C.); 2 Population Health Research Institute, St George’s University of London, Cranmer Terrace, Tooting, London SW17 0RE, UK; mussher@sgul.ac.uk; 3 Institute for Social Marketing, Faculty of Health Sciences and Sport, University of Stirling, Stirling FK9 4LA, UK; 4 National Centre of Smoking Cessation and Training, Dorchester DT1 2DY, UK; andy.mcewen@ncsct.co.uk; 5 Division of Epidemiology and Public health, University of Nottingham, Nottingham City Hospital, Nottingham NG5 1PB, UK; sarah.lewis@nottingham.ac.uk; 6 School of Health Sciences, University of East Anglia, Norwich NR4 7UL, UK; f.naughton@uea.ac.uk; 7 Usher Institute, College of Medicine and Veterinary Medicine, University of Edinburgh, Teviot Place, Edinburgh EH8 9AG, UK; lesley.sinclair@ed.ac.uk (L.S.); linda.bauld@ed.ac.uk (L.B.)

**Keywords:** smoking cessation, smoking, pregnancy, e-cigarettes, electronic cigarettes, stop smoking services, survey, interviews, mixed methods

## Abstract

Smoking in pregnancy remains a public health problem. In the UK e-cigarettes are the most popular aid to quitting smoking outside of pregnancy, but we don’t know the extent of e-cigarette use in pregnancy or how English Stop Smoking Services (SSS) respond to pregnant women who vape. In 2015 we surveyed SSS managers about cessation support for pregnant women and responses to clients who vaped. Subsequently we interviewed a sub-sample of managers to seek explanations for the SSS’ position on e-cigarettes; interviews were thematically analysed. Survey response rate was 67.8% (72/106); overall managers reported 2.2% (range 1.4–4.3%) of pregnant clients were using e-cigarettes. Most SSS reported supporting pregnant women who already vaped, but would not recommend e-cigarette use; for women that were still smoking and not using e-cigarettes, 8.3% of SSS were likely/very likely to advise using e-cigarettes, with 56.9% of SSS unlikely/very unlikely to advise using them. Fifteen respondents were interviewed; interviewees were generally positive about the potential of e-cigarettes for cessation in pregnancy although concerns about perceived lack of evidence for safety were expressed and most wanted research on this. Clear guidance on e-cigarette use informed by pregnancy specific research will assist SSS to provide consistent evidence-based support.

## 1. Introduction

Smoking when pregnant impacts on the health of mothers and unborn babies and also on children after birth and as they grow [1,2,3,4]. In England, currently around 10.8% of women are smoking at the time they have their babies, but in some areas this is much higher (e.g., 26% in Blackpool) [5]; rates are also much higher amongst younger and more deprived mothers [6]. Women are particularly motivated to quit smoking during pregnancy. Around half of those who smoke report quitting with most doing so just before or on discovering they are pregnant [6,7]; however, many find it difficult and try multiple times without success [8,9], and rates of decline in smoking at time of delivery appear to have stalled more recently [5]. Regrettably, most women who quit during pregnancy re-start smoking within 6 months of birth [10,11,12]; this has negative impacts on their health and increases their children’s risk of being exposed to second-hand smoke. Additionally, smokers’ children are more likely to start smoking themselves [13]. Pregnancy and the postpartum period, therefore, are crucially important times for helping women to quit as stopping for good improves both women’s and children’s health.

For non-pregnant smokers, e-cigarettes are now the most popular smoking cessation aid in England and are used in up to 40% of quit attempts [14]. Outside of pregnancy, increasing e-cigarette use might explain decreasing rates of SSS uptake and use of other pharmacotherapies like nicotine replacement therapy (NRT); monitoring data in non-specialist SSS suggests that few clients are using e-cigarettes [15] and as e-cigarettes mimic sensory aspects of smoking this may make them more appealing. There is some evidence that e-cigarettes work for smoking cessation [16] and in 2014, 16,000–22,000 smokers who would not otherwise have quit are estimated to have quit long-term after using e-cigarettes [17]. It is also possible that e-cigarettes could work for smoking cessation in pregnancy, and some pregnant women may already be using e-cigarettes for this, or to avoid postpartum smoking relapse [18]. Recently, authoritative UK groups, including Public Health England (PHE), the Royal College of Physicians, the Smoking in Pregnancy Challenge Group, and the National Centre for Smoking Cessation and Training (NCSCT) who provide training for all SSS staff, have stated that they believe benefits from e-cigarettes are likely to be far greater than harms, including in pregnancy [19,20,21,22,23]. However, safety concerns held by many women and health professionals may deter e-cigarette use and currently the prevalence of e-cigarette use in pregnancy and in the postpartum period is not known.

The World Health Organization recommends that all pregnant smokers should be routinely offered advice and psychosocial interventions for tobacco cessation [24]. In the UK, National Health Service (NHS) Stop Smoking Services (SSS) support smokers to quit, and the UK National Institute for Health and Clinical Excellence (NICE) guidance recommends that pregnant women who smoke are referred for such support [25]. Guidance is less specific, however, about how abstinent smokers should be supported after childbirth to avoid returning to smoking; only that relapse prevention should be offered [26], and that women require support “throughout pregnancy and beyond” whilst acknowledging that no interventions are of proven efficacy [25,27]. It is not clear how SSS have responded to this rather non-specific guidance on preventing return to smoking.

E-cigarette use in pregnancy could affect pregnant smokers’ use of NRT and perhaps of other interventions that SSS provide; NRT is offered to most pregnant women who attend SSS, but women already using e-cigarettes may be less likely to accept this. In addition, the recently published statements and guidance on e-cigarette use in pregnancy may be starting to influence SSS stance [19,20,21,22,23]. Consequently, this mixed method study using a survey of SSS managers followed by qualitative interviews, attempts to identify whether and if so, how, SSS have needed to adapt or respond to e-cigarette use amongst pregnant women. Specifically, we sought English SSS managers’ views on e-cigarette use amongst pregnant women who seek SSS support, the cessation interventions SSS currently offer, how SSS attempt to prevent women returning to smoking postpartum and their views on using e-cigarettes in this context.

## 2. Materials and Methods

### 2.1. Design

We undertook an explanatory sequential mixed methods design [28], with priority placed on a quantitative survey and where subsequent qualitative interviews were primarily used to help explain and investigate further the survey findings. Both sets of data were analysed separately in sequence, with the survey analysed first followed by the interviews, and the two approaches were mixed at the interpretation level.

### 2.2. Survey

#### 2.2.1. Identifying Survey Respondents

The survey was designed to be answered by SSS managers who were responsible for services to pregnant smokers. Since 1 April 2013, English Local Authorities (i.e., councils) rather than the health service have commissioned SSS and at the time of the survey there was no definitive list of managers of SSS that supported pregnant women. In some local authorities more than one local SSS to serve pregnant smokers may be commissioned and, in others, a local SSS may be commissioned but shared with a different local authority (i.e., one SSS may be commissioned by two different local authorities). Therefore, to determine the most appropriate people to complete the survey, we contacted local authority commissioners and asked them to confirm which SSS(s) they commissioned, and to provide us with the name and contact details of the individual(s) responsible for the day-to-day running of their SSS for pregnant women. Once the manager was identified, we contacted them by email or telephone to explain that we would like them to complete a survey, and to let them know what the survey was about. We asked them to confirm that they were most appropriate person to complete this or, if not, to send us the contact details of a more suitable person.

#### 2.2.2. Survey Dissemination and Completion

Once identified, an invitation email containing a link to the online survey [29] was sent to the SSS manager. To ensure that only one response was received for any SSS, we generated a unique link to one questionnaire for each service; if anyone managed more than one service they were sent two different links. All survey responses were collected over encrypted (SSL) connections, and managers were informed that their responses would remain confidential and non-identifiable. If they preferred, or were unable to access the online survey, managers were also given the option of filling in a paper or email version or to complete this by telephone. After 1 week, non-respondents were sent a reminder email. If there was still no response they were emailed again or followed up by telephone. The survey was available for completion between 24 August and 23 November 2015.

#### 2.2.3. Survey Design

The design and content of the survey were tested and piloted with two SSS managers (no changes were made) before distributing it to the remaining potential respondents. Items in the survey [full survey available online, Supplementary Materials, S1] related to the period between 1 April 2014 and 31 March 2015 and asked about SSS management of pregnant smokers. Topics included:

(a) *Scope of service*: number setting quit dates; if SSS targeted specific groups of pregnant smokers (e.g., under 18s, those in prison, with mental health problems, from deprived areas); whether SSS provided support to pregnant smokers who also vape (use e-cigarettes), to recent ex-smokers who do not vape and to recent ex-smokers who vape.
(b) *E-cigarettes in pregnancy*: number using “unlicensed nicotine containing products” (primarily e-cigarettes); how SSS practitioners would advise women about using e-cigarettes in three different clinical scenarios.
(c) *Other treatments for smoking cessation*: types of support provided; proportion of women who use each type; location and methods for delivering support; combinations of behavioural support and/or NRT offered (single therapy NRT was defined as prescribing only one NRT product at a time (either long or short acting) and dual therapy NRT as NRT patch plus a short-acting NRT product (e.g., gum, nasal spray, lozenge)); how long they thought women typically used NRT for when trying to stop smoking (pregnant women in NRT trials have generally shown low adherence to treatment [30]); if they advised a ‘cut down to quit’ approach in pregnancy (outside of pregnancy, NRT can be used to help people to cut down on the number of cigarettes they smoke before quitting). For the proportions of women using each treatment, SSS were asked to indicate whether or not the data provided was estimated.
(d) *Return to smoking after childbirth*: support provided to women in the period immediately after childbirth; type of treatments offered for this; views on using e-cigarettes to prevent return to smoking after childbirth.

### 2.3. Follow-up Interviews with Stop Smoking Service Managers

At the end of the survey, respondents were asked if they would be willing to be contacted to take part in a telephone interview to expand on their initial responses and to help identify future research needs. From those that agreed, we aimed to conduct interviews with approximately 16 SSS managers, selected to ensure a range of geographical locations, size and approach to treatment according to their initial survey responses. They were contacted by email, and a date arranged to conduct the interview. Interviews were conducted between May to July 2016 by R.W. and A.D., who introduced themselves as researchers from the University of Nottingham who worked outside the SSS organisation. SSS staff were asked to confirm whether they were the same person who had completed the survey and were asked to clarify their role in the SSS in relation to pregnant women. Participants were asked for verbal consent for the researchers to undertake and record the interview and were advised that neither individuals nor services would be identified in any written reports or presentations. Interviews lasted between 30–60 min, were audio recorded, anonymised and were transcribed verbatim.

Interviews were structured, and interviewees were asked about a range of issues relating to service provision in pregnancy including the service’s position on e-cigarettes, views around NRT adherence and advice, and issues around women returning to smoking postpartum. Regarding e-cigarettes, we aimed to gain further insight into what might hinder or facilitate services to recommend these in pregnancy. Thus we asked how services generally viewed e-cigarette use in pregnancy, whether this had changed recently, and if this was different for non-pregnant clients; what influenced or guided their position on e-cigarettes in pregnancy; what evidence or information they wanted to help guide their practice, and, again, if this was different for pregnant smokers compared to their non-pregnant clients.

### 2.4. Data Analysis

*Survey*: Statistical analyses were conducted using STATA 14 (StataCorp, College Station, TX, USA) [31]. Descriptive analyses were conducted to summarise the scope of services provided by SSS, how e-cigarettes were dealt with in SSS, provision of other treatments for smoking cessation in pregnancy and support provided for avoiding return to smoking after childbirth.

*Interviews*: NVivo 10 (QSR International, Melbourne, Australia) software was used to assist with the coding of the data. For this paper, we present only interview findings relating to e-cigarettes. Inductive thematic analysis, as described by Braun and Clarke [32] was used to identify and report patterns and themes within the text. Data were transcribed verbatim. S.C. and K.A.C. analysed the data independently; they read and re-read the interview transcripts and noted initial ideas and emerging themes to represent interviewees’ views on e-cigarettes in general, and on barriers against and facilitators for SSS recommending e-cigarettes to clients. The researchers then discussed their initial codes and themes, until they reached consensus and final themes were established. Key themes from the data are presented.

The work was conducted according to the tenets of the Declaration of Helsinki. The survey and interviews were conducted as a service evaluation and therefore ethical opinion was not required.

## 3. Results

### 3.1. Survey

We identified the commissioning leads with public health duties at 152 English local authorities and from these obtained contact details for the managers of 106 SSS. From the 106 SSS managers we received 72 replies (67.9% response rate).

#### 3.1.1. Scope of Services

The vast majority of SSS reported supporting any pregnant women who smoked, regardless of whether they also vaped (used e-cigarettes) with 94.4% (68/72) supporting pregnant smokers who also vaped. Fewer SSS reported supporting pregnant women who were recent ex-smokers (64.3% (45/72)) but again, women were supported irrespective of vaping with 61.9% (44/72) supporting recent ex-smokers who vaped.

Many reported that they also target specific groups of pregnant smokers including women under 18 years (59/72, 81.9%), those with mental health problems (52/72, 72.2%) and those from more deprived areas (64/72, 88.9%). Although not a predefined option, two SSS mentioned targeting black minority ethnic women. The mean number of pregnant women who set quit dates with SSS annually, indicating a services’ throughput or size, was 138.6 (SD 99.2), with a range across services of 11–391.

#### 3.1.2. Support Offered

The types of support offered by SSS during pregnancy are shown in Table 1. One-to-one support in clinic settings was the most common type of support with 93.1% of SSS offering this, and a median of 45% of women using this when available. One-to-one support in homes was also popular, being offered by 72.2% of SSS and used by 52% of women within these services. Telephone support was offered by 59.7% of SSS, but only taken up by 10%; text message support was offered by 40.3% and taken up by 5%. Email support was only offered by 15.3% of SSS and very few women were reported to use this type of support. Individual SSS reported very varied uptake across all types of support as demonstrated by the wide interquartile ranges (IQR) shown within Table 1.

All SSS offered NRT to pregnant smokers. The majority (*n* = 52, 72.2%) first offered either single or dual therapy depending on client preference, with 14 (19.4%) first offering dual therapy, and 6 (8.3%) single therapy. Over half of SSS (44, 61.1%) offered behavioural support without NRT, but they reported that on average only 10.5% of women chose this option. Just one SSS reported that they offered women NRT without expectation that they would attend for other support. The treatment option that managers reported to be most frequently accepted was behavioural support plus dual therapy NRT (70% of women (IQR 40–90%)); behavioural support with single therapy NRT was taken up by 30% of women (IQR 16–60%) (Table 1). Some SSS (14, 19.5%) reported advising using NRT to ‘cut down to quit’ in pregnancy, but the majority (47, 65.3%) were unlikely to recommend this. When asked about how long women tended to use NRT when trying to stop smoking, around half of SSS (38/72, 52.8%) reported that women were most likely to use NRT for at least 8 weeks, but 10 SSS (13.9%) reported that NRT was most commonly used for less than 4 weeks.

#### 3.1.3. E-Cigarettes in Pregnancy

Forty-two of the 72 SSS (58.3%) said they recorded the number of pregnant women reporting using e-cigarettes during their pregnancy, and 40 of these provided their figures. Overall, of the 5167 pregnant women setting a quit data across these 40 services, 116 (2.2%) were recorded as using e-cigarettes.; this varied from 1.4% (14/1014) in the South of England, 2.0% (39/1976) in the Midlands and East of England, 2.6% (48/1827) in the North of England, and 4.3% (15/350) of women in London.

Table 2 shows, for three clinical scenarios, how SSS believed their advisors should respond with respect to recommending e-cigarette use during pregnancy. Generally, regardless of the scenario, few SSS were likely to advise pregnant women to either start or continue using e-cigarettes. There were a variety of responses about how to approach the issue of e-cigarette use; many SSS reported being neither likely nor unlikely to advise pregnant women to use or continue to use e-cigarettes; this ranged between 31.9% and 36.1%, depending on the scenario.

For women that were still smoking and not using e-cigarettes, just 8.3% of SSS were likely or very likely to advise they use e-cigarettes, with 56.9% of SSS either unlikely or very unlikely to advise they use them. In comparison, if women had stopped smoking and were already using e-cigarettes, 19.5% of SSS were either likely or very likely to advise women to continue using e-cigarettes, with 43.0% of SSS either unlikely or very unlikely to advise they continue to use e-cigarettes. However, only a third of SSS (24, 33.3%) reported having a service-wide e-cigarette policy, and just 13 (18.1%) of these also had an e-cigarette policy specifically for pregnancy

#### 3.1.4. Return to Smoking after Childbirth

Most SSS (68, 94.4%) said that they aim to advise women routinely on avoiding returning to smoking after childbirth. However, only 43 of the 72 SSS (59.7%) reported that they contact women who stopped smoking in pregnancy in the postpartum period. SSS reported that they recommend or suggest a variety of different treatments to help prevent return to smoking after childbirth (Table 1). However, as in pregnancy, few SSS would advise women use e-cigarettes to help prevent return to smoking; just nine (12.5%) were likely or very likely to advise e-cigarettes for this (Table 2).

### 3.2. Interviews with Stop Smoking Service Managers

Of the 72 SSS managers who completed the survey, 33 indicated that they would be willing to be interviewed, 25 were invited by email and telephone interviews conducted with 15 SSS managers. From their survey responses, these covered a wide geographical spread, with three SSS in the North of England, five in the Midlands, four in the South and three in London. The number of pregnant smokers setting a quit date in these services during 2014–2015 ranged from 16 to 341 (mean 142.3, SD 99.5). Their responses to the questions about recommending use of e-cigarettes ranged from very likely to very unlikely. In addition to behavioural support, four SSS initially offered dual therapy NRT, two single therapy NRT and nine offered dual or single NRT depending on client preferences. Two SSS specifically mentioned providing support with e-cigarettes.

We identified three overarching themes related to the managers’ views on SSS offering e-cigarettes to pregnant women: general SSS stance on e-cigarettes use in pregnancy; factors that influence this position; and barriers to SSS in pregnancy becoming more ‘e-cigarette friendly’. According to NCSCT guidance, an ‘e-cigarette friendly’ SSS is one that ‘supports clients who want to use an e-cigarette to help them quit smoking and reaches out to smokers considering using an e-cigarette to come to the service for behavioural support’ [23]. The term ‘e-cigarette friendly’ was frequently used by interviewees so we accepted this at face value whenever it was mentioned. Additionally, in our analysis, we used this term to describe any service that indicated they actively welcomed and supported anyone who wanted to use e-cigarettes for quitting.

#### 3.2.1. General Stance on E-Cigarette Use in Pregnancy

The interviews suggested that the majority of SSS had had a clear and recent shift in stance on e-cigarettes in pregnancy; many services have already changed their position towards being e-cigarette friendly, while others were in the process of changing their approach or hoped to make this transition in the near future. A small proportion of managers noted they did not anticipate imminent changes towards e-cigarettes in pregnancy within their services.

The managers reported a spectrum of policies in operation that reflected their services’ stance. These ranged from currently being ‘e-cigarette friendly’ (*“we welcome it [using e-cigarettes to quit in pregnancy] and we’re very happy”; Participant ID—P’80*), through those that neither encouraged nor discouraged e-cigarette use (*“we decided to take the stance of using e-cigarette as a back-up option because at the end of the day it’s about patient choice”; P’79*), to those that would still give behavioural support to pregnant smokers who wanted to use e-cigarettes, but record it as being *“against medical advice” (P’72)* in their notes.

Even in the most e-cigarette friendly SSS, however, e-cigarettes were not usually seen as the first choice for pregnant women and with all pregnant women, including those who were already using e-cigarettes, managers reported they would still discuss licensed NRT products to *“make sure that they were fully informed when they made their decision” (P’73)*.

When asked for comparisons between offering e-cigarettes to the general population of smokers versus pregnant women there were notable differences, with services generally proceeding with more caution in pregnancy; some of the managers interviewed described the pregnancy specialist services to be the most reluctant to adopt e-cigarettes:

“(…) there’s a much greater risk isn’t there in terms of having a pregnant woman using them because it’s not just themselves that they’re harming but it’s potentially the foetus as well. So it’s you know, two lives versus one in a way”

*(P’79)*

“(…) in pregnancy, obviously we probably have a less of a—a less of a ‘oh yeah carry on, go ahead’ attitude”

*(P’82)*

“That [becoming an e-cigarette friendly service] was integrated within the main stop smoking service quite quickly and easily. There was some resistance within the smoking and pregnancy specialist service”

*(P’79)*

Generally, though, the attitude of many SSS was that if e-cigarettes could help women to stop smoking in pregnancy or help to prevent relapse afterwards, their use could be beneficial, particularly if other methods had failed, and was seen as *“harm reduction” (P’84)*:

“And for some women who’ve struggled with NRT have just, you know, haven’t managed to quit with that, this is another option to really use and to, to embrace really if that’s what they want to use. At least that gets them off the cigarettes, that’s really our view.”

*(P’83)*

#### 3.2.2. Factors that Influence E-Cigarette Stance

Various factors appeared to influence SSS stance or any shift in stance towards e-cigarettes, both in general and in pregnancy, including national and local policies, clinical experience and personal views.

(1) National Guidance and local commissioning policies

Many interviewees welcomed the recent national e-cigarette guidance, particularly that published by Public Health England (PHE) [19] and the National Centre for Smoking Cessation and Training (NCSCT) [22,23]. They felt that as this came from *“reliable”* and *“trustworthy”* sources, it had encouraged a positive shift in stance towards e-cigarettes in general:

“I think we’re much more positive than we were. I think initially four or five years ago it was, you know, quite frightening prospect for stop smoking services. (…) but over a period of time and particularly with sort of very reputable organisations like ASH, like the NCSCT, like Public Health England coming out with documents and position statements and policies, you know, sort of guidance, I think it’s, the position has moved an incredibly long way.”

*(P’87)*

Some also mentioned that training and personal conversations with representatives of the organisations issuing the guidance, including PHE and NCSCT, had helped further.

Local commissioning policies appeared to have more direct influence on services’ e-cigarette stance than national guidance. Previously SSS could make many of their own decisions on service delivery, but their stance was now directly affected by their local commissioning policies:

“… we have a very tightly commissioned service spec that allows us to do certain things and doesn’t allow us to do other things. So whereas in the past as a Service Manager there was quite a lot of leeway to improvise and innovate, I don’t have that anymore. So the position on e-cigarettes is a position that’s laid down by commissioners…”

*(P’75).*

According to the managers, many local commissioners seemed to have taken the national guidance on board, which in turn appeared to facilitate the adoption of a more e-cigarette friendly stance. For example one manager said that their Director of Public Health whose stance was previously cautious, became *“much more open to them [e-cigarettes] in an official sense” (P’72)* due to emerging evidence and guidance, which resulted in a shift towards e-cigarette friendly approach within the service they commissioned.

Some mangers, however, felt that commissioners’ attitudes had a negative impact on their services’ e-cigarette stance, and that they might act differently if they didn’t have these constraints:

“No, even with all the publications, they’re still not 100% convinced that e-cigs are safe or less harmful in pregnancy, so they won’t commission them or allow us to promote them, which I find very frustrating.”

*(P’74)*

Furthermore, some managers noted that national guidance had been received with scepticism by their commissioning bodies, some of whom preferred to go by NICE guidance [25] even though these were not recently updated, or by decisions made locally (e.g., from local Tobacco Control Alliances); interviewees felt this might be prone to individual opinions or biases:

“There was a lot of discussion around it [the PHE report [19]] and a lot of people had done research about exactly who produced that report and some of the actual research statistics that were used around that report so it didn’t get a good reception that report, there was a lot of doubt over the validity of it.”

*(P’75)*

This caused a lot of frustration in most of the services who weren’t yet e-cigarette friendly, particularly when they felt these decisions were being made by local politicians without relevant expertise in smoking cessation.

(2) Clinical experience

Seeing pregnant women successfully quit using e-cigarettes, with few negative side effects acted as a facilitator towards adopting a more e-cigarette friendly stance:

“it’s the clinical experience that has helped and seeing that women have managed to stay smoke free where they might not have done otherwise and they seem to do well and have a good outcome when the baby is born”

*(P’73)*

Unsurprisingly, SSS that discourage e-cigarette use in pregnancy had less encouraging evidence from practice at their disposal that would support e-cigarette use:

“So I can’t really draw on any experience that says ‘yeah, we’ve got people that are pregnant and are using them or they’re using them and they think they’re great, they’re using them and they don’t like them’ so I’m a bit in the dark on that.”

*(P’72)*

(3) Personal views

Managers of SSS that were not yet e-cigarette friendly often expressed personal views towards e-cigarette use in pregnancy that were more positive than the service’s official stance:

“I think you know if we accept that these are nicotine containing devices, we’re quite happy to proceed with nicotine replacement therapy, so we shouldn’t be so jumpy about using e-cigarettes in pregnancy.”

*(P’72)*

One interviewee, with a strong positive personal view on e-cigarettes felt it was her responsibility to at least encourage women to explore information on e-cigarettes, even though her service did not officially support e-cigarette use in pregnancy:

“I feel that I have a duty of care to give my women information, so I direct them to the Public Health England report and say to them that this is what Public Health England said, you can look it up, you can read it on your Internet for yourself, but at this moment in time I cannot recommend them.”

*(P’74)*

#### 3.2.3. Barriers to Stop Smoking Services in Pregnancy Becoming More ‘E-Cigarette Friendly’

Despite the fact that the majority of the managers were open to the idea of allowing e-cigarette use in general and with pregnant women and had largely e-cigarette-friendly attitudes, it was evident that they still had many reservations about e-cigarette safety and effectiveness in pregnancy. They noted a number of barriers that need to be overcome before e-cigarettes can be fully integrated into specialist smoking cessation services in pregnancy; these relate to evidence base on e-cigarettes, availability of some e-cigarettes as licenced medicinal products, and delivering a consistent message on e-cigarettes throughout antenatal services:

(1) Evidence

The need to improve the evidence base on the effectiveness and safety of e-cigarettes in pregnancy was apparent in the majority of responses. Many noted that robust and consistent evidence from reputable teams of academics and researchers in the field of smoking cessation, based on large cohort longitudinal studies and/or randomised controlled trials would be key to helping commissioners, managers, advisors and general public to understand the long-term effects of using e-cigarettes and, if the evidence confirmed it, to accept e-cigarettes as an appropriate quit aid and harm reduction tool:

“I suppose my reservations around them are there’s still insufficient evidence about the long term effects of use of them.”

*(P’75)*

“But I think what we would need is that kind of study, two or three perhaps different studies that actually come out and investigate what is the usage of e-cigarettes in pregnancy, how effective are they, do they help with cessation or with harm reduction and can we see any potential issues that would give us cause to say ‘no, we don’t think we can advocate these in pregnancy’. If I was to guess I’d probably think they’d come out in a positive light. So yeah, it’s that kind of a study that’s got academic rigour behind it or maybe it is authored by one of the luminaries within the stop smoking world”

*(P’72)*

In the vast majority of responses, the specific issues relating to pregnancy, such as safety of the unborn baby, were of paramount importance, and the evidence was considered to be particularly lacking in this respect. Therefore, the managers felt that research specific to pregnancy was essential, to help health professionals and commissioners understand how vaping affects birth outcomes as well as quit outcomes, when compared to smoking and abstinence from nicotine:

“I think we’d like to know how, in wider numbers, I mean obviously our numbers are fairly small, so it would be good to know across the country what different outcomes we’re seeing once the baby is born. How does it affect birth weight and the general health of the baby? Is there a consistent picture of babies born to women who are vaping being on a par with women who have gone completely smoke free? That would be useful. The potential impact would be helpful to know.”

*(P’73)*

Managers were also unsure about the product itself—many noted that there were gaps in the SSS knowledge on the specific properties of each brand/generation of e-cigarettes. They felt this was problematic and there was an apparent need for the advisors to understand these differences better in order to be able to recommend e-cigarettes which are the safest for pregnant women:

“‘Cause at the moment the Smoke Free services are not experts in electronic cigarettes and I think that’s sometimes a bit difficult because as a specialist service or specialist advisors we would normally know exactly what we could recommend. And having this kind of unknown product that we know that people are wanting to use, not having a great deal of understanding about how they work or how strong they are or which types of devices are the best in terms of nicotine delivery and nicotine treatment (…) And it is a bit complicated, if you’re not sure you’re giving the best advice and you don’t have any knowledge of how they’re working.”

*(P’79)*

In spite of NRT being commonly prescribed in pregnancy for over 10 years now, some were also still expressing doubts over the safety of prescribing nicotine in pregnancy:

Obviously we all know that if people can give up smoking and using nicotine then that’s absolutely the best for them and I think in pregnancy we still, we’re pretty sure that it’s not going to harm, nicotine is not going to harm the baby, but we’re not 100% sure. So I have reservations about the use of e-cigarettes in pregnancy at the moment. I don’t think there’s enough research been done.”

*(P’75)*

(2) Licencing of e-cigarettes

In the UK, all e-cigarettes are currently purchased as consumer products rather than prescribed or issued by health professionals as medicines, because although it is possible to get them formally licensed as medicines, as yet there aren’t any licensed products available. Some of the managers of services that did not fully subscribe to the e-cigarette friendly approach noted that this was a barrier to their service embracing them as a quit aid in pregnancy. They highlighted the need for the products to be regulated and ‘properly licensed’:

“P: We don’t actively encourage pregnant women to use e-cigarettes. We still tell the hospital if they are on e-cigarettes to refer them through to us and we use registered NRT to try and get them off e-cigarettes.

I: Ok and why does your service have this particular view or take this approach?

P: Well just because they’re not licensed really, they’re not licensed for use. So we can’t obviously advise pregnant women that they’re safe to use because we don’t know that they are.“

*(P’88)*

“I would like to see a properly licenced e-cigarette that we could use as a medication, as a product, that we were really, really confident wasn’t going to harm our clients in any way and one that was also licenced for use in pregnancy.”

*(P’75)*

(3) Consistent messages, training and implementation

The interviewees also noted that in order to successfully adopt e-cigarettes as a smoking cessation tool for pregnant women, there has to be a consistency across the antenatal services and specialist SSS throughout the country. This could ensure that the same clear messages about safety and effectiveness are delivered to all pregnant women, reducing the risk of miscommunication and confusion:

“(…) it comes down to specialist service advisors being able to impart that information but also for the midwifery team who are doing the carbon monoxide readings in the hospital and end up doing the referrals, that they also have the correct information to be able to pass onto pregnant women about the potential benefits and risks of e-cigarettes.”

*(P’79)*

“our two specialist advisors have done quite a bit of training with midwives to make sure that everybody understands the same thing and are certainly giving a consistent message to the women.”

*(P’73)*

## 4. Discussion

Overall there was no evidence that Stop Smoking Services had noticeably altered the support they provided to pregnant women following the emergence of e-cigarettes; the survey found that SSS rarely encountered pregnant women who used e-cigarettes, and the kinds of support they offered most frequently had not changed substantially since 2010–2011 [33]. Most SSS supported pregnant women who vaped, but there was much uncertainty about whether e-cigarettes should be used in pregnancy at all and few SSS actively recommended their use. SSS managers were much more positive about the potential use of e-cigarettes in pregnancy when they were interviewed, which was after Public Health England and others released guidance on e-cigarettes [19,20,21,22,23]. Nevertheless, managers still wanted more pregnancy-specific evidence on the safety of e-cigarettes during pregnancy, for e-cigarettes licensed as medications to be available, and for clear and consistent messages on e-cigarettes to be given to pregnant women whenever they received healthcare.

The study had a number of strengths, as well as some limitations. We identified a SSS and/or SSS manager for most areas of England, although there may have been SSS in other areas, so findings may not be representative of all services. However, by identifying services via commissioners, we took care to ensure that surveys were completed by those most knowledgeable about the local provision, and received responses from almost 70% of the services we contacted. Data were self-reported, and due to re-organisation, were occasionally for part years or estimated, but we believe that SSS managers would have taken care to provide the best information possible. As with all surveys, some questions may have been misinterpreted, but we piloted the survey with two SSS managers before distributing to the remainder, and this did not indicate any issues. We also provided additional information for questions where appropriate; for example, a screenshot of their database field for “unlicensed nicotine products”, and a definition of single and dual therapy NRT.

A particular strength of this study is that, to our knowledge, no others have investigated how specialist smoking cessation services have attempted to address e-cigarette use in pregnancy and postpartum. It was further strengthened by conducting follow up interviews with some services to gain more insight to their responses.

It is notable that in 2014–2015, SSS were encountering few pregnant women who reported using e-cigarettes, in spite of them being the most popular aid for smoking cessation amongst non-pregnant smokers during this period [14]. There could be various explanations for this, for example, pregnant women using e-cigarettes may have already quit, or perhaps they don’t seek help and instead try to quit without support from SSS. Alternatively, they may be less likely to use e-cigarettes perhaps due to safety concerns [18,34], or women may not admit to using them during pregnancy perhaps because of stigma [18]. Interestingly, a study of general (rather than specialist) English SSS reported comparable e-cigarette figures to those from our survey for all clients attending SSS during the same period, and were similarly reluctant to advise e-cigarette use [15].

Our survey found a lot of uncertainty amongst SSS managers about whether to advise pregnant women to use e-cigarettes, and how to help women postpartum to prevent return to smoking. This is noteworthy as SSS managers are likely be amongst the best informed about the latest recommendations for smoking cessation; although we didn’t ask about training or education in the survey, NICE, PHE and the Department of Health all recommend that anyone who helps people to quit should be NCSCT certified, and NCSCT provide general and pregnancy specific smoking cessation training free of charge. Commissioners of SSS insist that all advisors undertake this training. NCSCT also recommend that managers and commissioners complete the training. Irrespective of clinical scenario, few SSS were likely to advise e-cigarette use, but some of these survey responses are difficult to interpret. For example, where a SSS indicated they were unlikely to advise women to continue using e-cigarettes, we don’t know if they actively discouraged their use or if they did not address the issue at all (e.g., said nothing); however, our subsequent interviews indicate that overall this was more likely to be the latter. Most SSS did not have a formal policy regarding use in pregnancy; this, and their uncertainty about how they advise on e-cigarettes, is perhaps unsurprising, as there is currently no NICE guidance for e-cigarettes in pregnancy, and no specific NICE recommendations for postpartum relapse prevention other than providing ‘ongoing support’ and warning against second-hand smoke exposure [10,25]. Although UK guidance or statements from the Smoking in Pregnancy Challenge Group, PHE, the Royal College of Physicians, and the NCSCT, endorse vaping amongst pregnant women who are unable or unwilling to quit smoking using traditional methods, most had only recently been published or have been published since this study [19,20,21,22,23].

In the U.S., surveys of health professionals caring for pregnant women have indicated that many appear to have concerns about the use of e-cigarettes; obstetricians and gynecologists were uncertain about how to advise on e-cigarettes due to lack of guidance [35], and of US family physicians who provided obstetric care, most respondents thought that e-cigarettes are unsafe to use during pregnancy [36]. Many US quitline professionals also have negative beliefs about e-cigarettes outside of pregnancy [37]. Our interviewees did not appear to have the same level of apprehension as those seen in the US studies; many told us that their SSS were starting to adopt a more positive attitude towards e-cigarettes in pregnancy, and that recent guidance had influenced them. However, several felt that some SSS commissioners had not yet taken this on board, and this was one of the main reasons for not recommending e-cigarettes, although many interviewees also said that they would like more specific evidence in pregnancy.

Some of our findings are similar to those seen outside of pregnancy; a survey of SSS practitioners wanted more evidence and licensed products before recommending them [15]. Interviews with generic SSS staff also show that they have faced challenges similar to the ones we identified for pregnancy specific services to becoming more e-cigarette friendly, including the role of local public health commissioners [38]. However, as more guidance has become available, commissioner attitudes may have started to change, at least for general smokers. A recent survey of tobacco control leads found that 75% of respondents said their SSS now supported smokers who want to use e-cigarettes to aid their quit attempt, and none reported discouraging e-cigarette use although questions were framed differently to our survey and were aimed at general SSS rather than pregnancy specific SSS [39].

Since 2010–2011 there has been a steady decline in the number of pregnant women setting quit dates with SSS, mirroring the data seen for all smokers [40], and it has been suggested that e-cigarettes are a key reason for decline in general SSS attendance [15]. However, in spite of this, overall rates of smoking at time of delivery continues to fall in England; 13.7% of women reported smoking at delivery in 2010–2011, whereas this was 10.8% in 2017–2018, but whether any of this fall is due to women quitting using e-cigarettes is unknown [5]. Our survey gives a snapshot for one year at a time of great change amongst SSS; cuts to smoking cessation budgets had been reported in 39% of local authorities, with over half of services having some form of recommissioning or reconfiguration [41,42]. Therefore, it is interesting that in spite of these changes in organisation, and the widespread availability of e-cigarettes, the type of support offered had changed little since 2010/2011 [33]. As many SSS face further cuts this may not continue; in 2017, 74% of local authorities reported still having some form of specialist SSS (e.g., for pregnant smokers) [39].

It is possible that women may find e-cigarettes more satisfying than NRT and so be more likely to use them, but there may also be ways to encourage women to use NRT more effectively. For example, as safety of both is likely to be a concern to pregnant women in particular [43], informing them of the relative harms of both NRT and e-cigarettes compared with cigarettes needs to be more widely disseminated, particularly by health professionals. From the data provided, it appears that SSS have few concerns about supplying dual NRT despite little evidence on safety or effectiveness, and yet they had a much more cautious approach to e-cigarettes. Currently only one-third of smokers who do not use e-cigarettes believe they are less harmful than cigarettes [14], and so pregnant women may be discouraged from using them due to perceived safety issues. Surveys and qualitative research on e-cigarettes amongst pregnant women indicate that they generally perceive e-cigarettes to be safer than smoking, but they have uncertainty about absolute safety [18,34,44,45,46] and some women reported getting mixed messages from health professionals [34]. Although many had tried e-cigarettes, few women reported using them during pregnancy [47,48,49,50,51].

Now that guidance, particularly in the UK, is more positive about use of e-cigarettes for smoking cessation, including in pregnancy, some SSS are starting to feel more confident in providing advice on their use, but there are still inconsistencies and many still express uncertainties. As there is still a lack of specific evidence in this area, more research, particularly on safety (including harms relative to smoking and to NRT treatment) and effectiveness in pregnancy is needed; at least one randomized controlled trial is currently underway [52]. Findings from this research and other studies in pregnancy and postpartum will assist SSS and other health professionals to tailor their advice.

## 5. Conclusions

Few pregnant women accessing SSS were using e-cigarettes, and overall SSS were unlikely to advise e-cigarette use, although following publication of guidance this appears to be changing. There is no evidence that e-cigarette use in pregnancy is affecting the type of support provided by SSS.

However, the evolving landscape for e-cigarettes leaves some SSS more likely to advise or encourage their use in pregnancy, whilst others remain unlikely to advise their pregnant clients to do so. More pregnancy specific evidence on e-cigarette safety and effectiveness to inform official guidance, should help to ensure consistent messages across all SSS and other maternity services.

## Figures and Tables

**Table 1:** Types of support offered by Stop Smoking Services (SSS) during pregnancy, and support suggested for preventing return to smoking postpartum.

Support Provided during Pregnancy	Services Offering this Type of Support *N* (%)	% of Women within these Services Using this Type of Support Median (Interquartile Range)
Number of Stop Smoking Services	*N* = 72	
Support type/location		
One-to-one support in women’s homes	52 (72.2)	52 (10–85)
One-to-one support in a clinic setting	67 (93.1)	45 (15–90)
Telephone support	43 (59.7)	10 (5–25)
Text message support	29 (40.3)	5 (1–75)
Email support	11 (15.3)	0 (0–3.4)
Other	17 (23.6)	5.5 (0.5–30)
Interventions		
Behavioural support plus single therapy NRT	56 (77.8)	30 (15.7–60)
Behavioural support plus dual therapy NRT	61 (84.7)	70 (40–90)
Single therapy NRT only (no expectation that women attend for other support)	1 (1.4)	5
Dual therapy NRT only (no expectation that women attend for other support)	1 (1.4)	5
Behavioural support only	44 (61.1)	9 (4.8–14.5)
Other	11 (15.3)	1.7 (0.03–6)
**How is NRT first offered?**	**Services *N* (%)**	
Single therapy	6 (8.3)	n/a
Dual therapy	14 (19.4)	n/a
Single or dual (client preference)	52 (72.2)	n/a
**Does SSS routinely make contact postpartum with women who stopped smoking in pregnancy?**	**Services *N* (%)**	
Yes	43 (59.7)	n/a
No	28 (38.9)	n/a
Missing	1 (1.4)	n/a
**Does SSS routinely advise pregnant women on avoiding relapse after childbirth?**	**Services *N* (%)**	
Yes	68 (94.4)	n/a
No	3 (4.2)	n/a
Missing	1 (1.4)	n/a
**Support for preventing return to smoking postpartum**	**Services recommending this support *N* (%)**	
Single therapy NRT (patch)	22 (30.6)	n/a
Single therapy NRT (short-acting)	31 (43.1)	n/a
Dual therapy NRT (patch + short acting)	26 (36.1)	n/a
Varenicline	9 (12.5)	n/a
Buproprion	2 (2.8)	n/a
Behavioural support	54 (75.0)	n/a
Self-help support	29 (40.3)	n/a
E-cigarettes	6 (8.3)	n/a
Other	9 (12.5)	n/a

NRT: Nicotine Replacement Therapy; n/a: not applicable.

**Table 2:** E-cigarette advice given by pregnancy stop smoking practitioners: responses to different scenarios in pregnancy and postpartum.

Number of Services (%)	Very Unlikely to Advise Using/Continue Using E-Cigarettes	Unlikely to Advise Using/Continue Using E-Cigarettes	Neither Likely or Unlikely to Advise Using/Continue Using E-Cigarettes	Likely to Advise Using/Continue Using E-Cigarettes	Very Likely to Advise Using/Continue Using E-Cigarettes	Missing
How do stop-smoking practitioners respond to pregnant women who are smoking but not using e-cigarettes, and who ask whether or not it would be a good idea to use them?	26 (36.1)	15 (20.8)	23 (31.9)	6 (8.3)	0	2 (2.8)
How do stop smoking practitioners generally respond to pregnant women who smoke and are using e-cigarettes?	19 (26.4)	18 (25.0)	24 (33.3)	9 (12.5)	1 (1.4)	1 (1.4)
How do stop smoking practitioners generally respond to pregnant women who have stopped smoking and are using e-cigarettes?	16 (22.2)	15 (20.8)	26 (36.1)	11 (15.3)	3 (4.2)	1 (1.4)
What is your SSS view on using e-cigarettes to prevent relapse after childbirth?	13 (18.1)	15 (20.8)	34 (47.2)	7 (9.7)	2 (2.8)	1 (1.4)

## Supplementary Materials

The following is available online at http://www.mdpi.com/1660-4601/16/1/110/s1, S1: survey of local Stop Smoking Service support for pregnant women.

## References

[1] Cnattingius S. (2004). The epidemiology of smoking during pregnancy: Smoking prevalence, maternal characteristics, and pregnancy outcomes. Nicotine Tob. Res.

[2] Gluckman P.D., Hanson M.A., Cooper C., Thornburg K.L. (2008). Effect of in utero and early-life conditions on adult health and disease. N. Engl. J. Med..

[3] Delpisheh A., Kelly Y., Rizwan S., Attia E., Drammond S., Brabin B.J. (2007). Population attributable risk for adverse pregnancy outcomes related to smoking in adolescents and adults. Public Health.

[4] Clifford A., Lang L., Chen R. (2012). Effects of maternal cigarette smoking during pregnancy on cognitive parameters of children and young adults: A literature review. Neurotoxicol. Teratol..

[5] NHS Digital Statistics on Women’s Smoking Status at Time of Delivery, England—April 2017 to March 2018. https://files.digital.nhs.uk/D2/C2C76A/stat-wome-smok-time-deli-eng-q4-17-18-rep.pdf.

[6] McAndrew F., Thompson J., Fellows L., Large A., Speed M., Renfrew M.J. Infant Feeding Survey 2010. https://files.digital.nhs.uk/publicationimport/pub08xxx/pub08694/infant-feeding-survey-2010-consolidated-report.pdf.

[7] Orton S., Bowker K., Cooper S., Naughton F., Ussher M., Pickett K.E., Leonardi-Bee J., Sutton S., Dhalwani N.N., Coleman T. (2014). Longitudinal cohort survey of women’s smoking behaviour and attitudes in pregnancy: Study methods and baseline data. BMJ Open.

[8] Pickett K.E., Wakschlag L.S., Dai L., Leventhal B.L. (2003). Fluctuations of maternal smoking during pregnancy. Obstet. Gynecol..

[9] Cooper S., Orton S., Leonardi-Bee J., Brotherton E., Vanderbloemen L., Bowker K., Naughton F., Ussher M., Pickett K.E., Sutton S. (2017). Smoking and quit attempts during pregnancy and postpartum: A longitudinal UK cohort. BMJ Open.

[10] Hajek P., Stead Lindsay F., West R., Jarvis M., Hartmann-Boyce J., Lancaster T. (2013). Relapse prevention interventions for smoking cessation. Cochrane Database Syst. Rev..

[11] Fingerhut L.A., Kleinman J.C., Kendrick J.S. (1990). Smoking before, during, and after pregnancy. Am. J. Public Health.

[12] McBride C.M., Pirie P.L., Curry S.J. (1992). Postpartum relapse to smoking: A prospective study. Health Educ. Res..

[13] Leonardi-Bee J., Jere M.L., Britton J. (2011). Exposure to parental and sibling smoking and the risk of smoking uptake in childhood and adolescence: A systematic review and meta-analysis. Thorax.

[14] West R., Beard E., Brown J. Trends in Electronic Cigarette Use in England. http://www.smokinginengland.info/sts-documents/.

[15] Hiscock R., Bauld L., Arnott D., Dockrell M., Ross L., McEwen A. (2015). Views from the Coalface: What Do English Stop Smoking Service Personnel Think about E-Cigarettes?. Int. J. Environ. Res. Public Health.

[16] Hartmann-Boyce J., McRobbie H., Bullen C., Begh R., Stead L.F., Hajek P. (2016). Electronic cigarettes for smoking cessation. Cochrane Database Syst. Rev..

[17] West R., Shahab L., Brown J. (2016). Estimating the population impact of e-cigarettes on smoking cessation in England. Addiction.

[18] Bowker K., Orton S., Cooper S., Naughton F., Whitemore R., Lewis S., Bauld L., Sinclair L., Coleman T., Dickinson A. (2018). Views on and experiences of electronic cigarettes: A qualitative study of women who are pregnant or have recently given birth. BMC Pregnancy Childbirth.

[19] McNeill A., Brose L.S., Calder R., Hitchman S.C., Hajek P., McRobbie H. (2015). E-Cigarettes: An Evidence Update.

[20] Royal College of Physicians (2016). Nicotine without Smoke: Tobacco Harm Reduction.

[21] Smoking in Pregnancy Challenge Group Use of Electronic Cigarettes in Pregnancy, A Guide for Midwives and Other Healthcare Professionals. http://smokefreeaction.org.uk/wp-content/uploads/2017/06/eCigSIP.pdf.

[22] McEwen A., Bauld L., Hermon Y. (2015). Smoking Cessation: A Briefing for Midwifery Staff.

[23] McEwen A., McRobbie H. (2016). Electronic Cigarettes: A Briefing for Stop Smoking Services.

[24] World Health Organization (2013). WHO Recommendations for the Prevention and Management of Tobacco Use and Second-Hand Smoke Exposure in Pregnancy.

[25] National Institute for Health and Clinical Excellence (2010). Smoking: Stopping in Pregnancy and after Childbirth (PH26).

[26] Samet J.M., Yoon S.Y., World Health Organization (2010). Chapter 9. Pregnancy and Postpartum Smoking Cessation. Gender, Women, and the Tobacco Epidemic.

[27] Myers K., West O., Hajek P. Rapid Review of Interventions to Prevent Relapse in Pregnant Ex-Smokers. Report to National Institute for Health and Clinical Excellence. https://www.nice.org.uk/guidance/ph26/evidence/rapid-review-of-interventions-to-prevent-relapse-in-pregnant-exsmokers-376427053.

[28] Creswell J.W., Plano Clark V.L. (2011). Chapter 3. Choosing a Mixed Methods Design. Designing and Conducting Mixed Methods Research.

[29] Online Surveys. https://www.onlinesurveys.ac.uk/.

[30] Coleman T., Chamberlain C., Davey M.-A., Cooper Sue E., Leonardi-Bee J. (2015). Pharmacological interventions for promoting smoking cessation during pregnancy. Cochrane Database Syst. Rev..

[31] StataCorp (2015). Stata Statistical Software: Release 14.

[32] Braun V., Clarke V. (2006). Using thematic analysis in psychology. Qual. Res. Psychol..

[33] Fahy S.J., Cooper S., Coleman T., Naughton F., Bauld L. (2014). Provision of smoking cessation support for pregnant women in England: Results from an online survey of NHS stop smoking services for pregnant women. BMC Health Serv. Res..

[34] Fallin A., Miller A., Assef S., Ashford K. (2016). Perceptions of Electronic Cigarettes among Medicaid-Eligible Pregnant and Postpartum Women. J. Obstet. Gynecol. Neonatal Nurs..

[35] England L.J., Anderson B.L., Tong V.T.K., Mahoney J., Coleman-Cowger V.H., Melstrom P., Schulkin J. (2014). Screening practices and attitudes of obstetricians-gynecologists toward new and emerging tobacco products. Am. J. Obstet. Gynecol..

[36] Northrup T.F., Klawans M.R., Villarreal Y.R., Abramovici A., Suter M.A., Mastrobattista J.M., Moreno C.A., Aagaard K.M., Stotts A.L. (2017). Family Physicians’ Perceived Prevalence, Safety, and Screening for Cigarettes, Marijuana, and Electronic-Nicotine Delivery Systems (ENDS) Use during Pregnancy. J. Am. Board Fam. Med..

[37] Cummins S., Leischow S., Bailey L., Bush T., Wassum K., Copeland L., Zhu S.-H. (2016). Knowledge and beliefs about electronic cigarettes among quitline cessation staff. Addict. Behav..

[38] Farrimond H., Abraham C. (2018). Developing E-cigarette friendly smoking cessation services in England: Staff perspectives. Harm Reduct. J..

[39] Cancer Research UK, Action on Smoking and Health Feeling the Heat: The Decline of Stop Smoking Services in England. Findings from a Survey of Local Authority Tobacco Control Leads. https://www.cancerresearchuk.org/sites/default/files/la_survey_report_2017.pdf.

[40] NHS Digital Statistics on NHS Stop Smoking Services in England—April 2017 to March 2018. https://files.digital.nhs.uk/CC/D8DC38/stat-stop-smok-serv-eng-q4-1718-rep.pdf.

[41] Anderson W., Cheeseman H., Commissioned by Cancer Research UK Reading between the Lines. Results of a Survey of Tobacco Control Leads in Local Authorities in England. https://www.cancerresearchuk.org/sites/default/files/reading_between_the_lines_-_tobacco_control_in_england_january_2016.pdf.

[42] Action on Smoking and Health, Commissioned by Cancer Research UK Cutting Down: The Reality of Budget Cuts to Local Tobacco Control. https://www.cancerresearchuk.org/sites/default/files/local_authority_survey_2016_report_cruk_finalfinal.pdf.

[43] Bowker K., Campbell K.A., Coleman T., Lewis S., Naughton F., Cooper S. (2015). Understanding pregnant smokers’ adherence to nicotine replacement therapy during a quit attempt: A qualitative study. Nicotine Tob. Res..

[44] Kahr M.K., Padgett S., Shope C.D., Griffin E.N., Xie S.S., Gonzalez P.J., Levison J., Mastrobattista J., Abramovici A.R., Northrup T.F. (2015). A qualitative assessment of the perceived risks of electronic cigarette and hookah use in pregnancy. BMC Public Health.

[45] England L.J., Tong V.T., Koblitz A., Kish-Doto J., Lynch M.M., Southwell B.G. (2016). Perceptions of emerging tobacco products and nicotine replacement therapy among pregnant women and women planning a pregnancy. Prev. Med. Rep..

[46] Wigginton B., Gartner C., Rowlands I.J. (2016). Is It Safe to Vape? Analyzing Online Forums Discussing E-Cigarette Use during Pregnancy. Womens Health Issues.

[47] Mark K.S., Farquhar B., Chisolm M.S., Coleman-Cowger V.H., Terplan M. (2015). Knowledge, Attitudes, and Practice of Electronic Cigarette Use Among Pregnant Women. J. Addict. Med..

[48] Ashford K., Wiggins A., Butler K., Ickes M., Rayens M.K., Hahn E. (2016). E-Cigarette Use and Perceived Harm among Women of Childbearing Age Who Reported Tobacco Use during the Past Year. Nurs. Res..

[49] Wagner N.J., Camerota M., Propper C. (2017). Prevalence and Perceptions of Electronic Cigarette Use during Pregnancy. Matern. Child Health J..

[50] Oncken C., Ricci K.A., Kuo C.-L., Dornelas E., Kranzler H.R., Sankey H.Z. (2017). Correlates of Electronic Cigarettes Use before and during Pregnancy. Nicotine Tob. Res..

[51] Kurti A.N., Redner R., Lopez A.A., Keith D.R., Villanti A.C., Stanton C.A., Gaalema D.E., Bunn J.Y., Doogan N.J., Cepeda-Benito A. (2017). Tobacco and nicotine delivery product use in a national sample of pregnant women. Prev. Med..

[52] Walton R., Przulj D., Naughton F., Myers Smith K., Cooper S., Sinclair L., McRobbie H., Manyonda I., Bauld L., Ussher M. HTA Project 15/57/85—Helping Pregnant Smokers Quit: Multi-Centre RCT of Electronic Cigarettes vs. Usual Care. https://www.journalslibrary.nihr.ac.uk/programmes/hta/155785#/.

