# Supplementary material for: Attitudes to E-Cigarettes and Cessation Support for Pregnant Women from English Stop Smoking Services: A Mixed Methods Study
Source: Int J Environ Res Public Health. 2019 Jan 3;16(1):110. doi: 10.3390/ijerph16010110 (PMC6338976; doi:10.3390/ijerph16010110)
Supplement: Supplementary file 1 [file ijerph-16-00110-s001.pdf]

Survey of local Stop Smoking Service support for pregnant women

Page 1: Introduction

The Smoking in Pregnancy research group at the University of Nottingham is interested in the role of e-cigarettes and NRT use in pregnancy, and on relapse to smoking after childbirth. We would like to find out how local Stop Smoking Services (SSS) currently deal with these issues to help ensure we conduct research that is relevant to the needs of SSSs and the NHS. We are therefore undertaking this survey to collect information about this. We will make these findings available to SSSs and also hope to publish them as a service evaluation.

Who should complete this survey

Managers who are responsible for services to pregnant smokers.

Questions in the survey are intended to be answered by people who oversee the day to day running of local SSS ***for pregnant smokers*** *(SSSP)*; effectively these people are service managers, but they may have quite varied titles (e.g. smoking cessation / tobacco control coordinators). It is very important that surveys are answered by these individuals because they are most likely to have detailed knowledge of how the SSS actually operates and how the support that the SSS can offer to pregnant smokers is actually delivered.

We are particularly interested to find out how your SSS supports **pregnant**

smokers and in particular what your SSS view and practices are regarding **e-cigarettes** used **in pregnancy**.

Unless stated otherwise, all questions refer to **pregnant smokers** and to the 12 month period between **1 April 2014 and 31 March 2015**.

How do I complete the survey?

There are 20 questions and progress through the survey is shown at the top of each page. If you are not able to complete this all in one sitting, you can click 'Finish later' at the bottom of the page. This will save your answers and you will then be able to close your browser and return to complete it later. This might be useful if, for example, you need to refer to other sources or people. If you *do* need to return to the survey at a later time you will need to use the link generated when you click 'Finish later'. Please note, we will not be able to see any of your answers until you click 'Finish' at the very end of the survey.

Queries

If you have any queries relating to this survey, please contact: Naomi Coleman-Haynes (Research Administrator) on 0115 7484040, email: naomi.coleman-haynes@nottingham.ac.uk, or Sue Cooper (Principal Research Fellow) on 0115 8231898, email: sue.cooper@nottingham.ac.uk

What topics are covered in the survey?

Section A: Scope of service
This section asks about the focus of your SSS and the kinds of *pregnant women* to whom you deliver support.

Section B: E-cigarettes in pregnancy
This section asks for further details about how the issue of e-cigarette use in pregnancy is dealt with by your SSS.

Section C: Other treatments for smoking cessation in pregnancy
This section asks for information about the kinds of support provided by your SSS between 1 April 2014 and 31 March 2015.

Section D: Relapse after childbirth

This final section deals with the period immediately after childbirth when many pregnant women might re-start smoking. We are interested to know whether your SSS provides support to women at this time.

Page 2: Stop Smoking Service name

1 Stop Smoking Service name: ✱ *Required*

More info

Page 3: Section A: Scope of service

This section asks about the focus of your SSS and the kinds of *pregnant women* to whom you deliver support

2 Which kinds of **pregnant women** does your SSS support? (select all that apply)

- ☐ Smokers who do not vape (i.e. do not use e-cigarettes)
- ☐ Smokers who also vape
- ☐ Recent ex-smokers (i.e. stopped since becoming pregnant or soon before pregnancy) who do not vape
- ☐ Recent ex-smokers who vape
- ☐ Other

2.a If you selected Other, please specify:

3 Does your service treat or target specific groups of **pregnant smokers**? (select all that apply)

- ☐ Women under 18 years
- ☐ Women in prison
- ☐ Women with mental health problems
- ☐ Women from more deprived areas
- ☐ Other

3.a If you selected Other, please specify:

4 How many **pregnant smokers** set a quit date with your service in the year 1 April 2014 to 31 March 2015?

Page 4: Section B: E-cigarettes in pregnancy

This section asks for further details about how the issue of e-cigarette use in pregnancy is dealt with by your SSS.

5 Stop Smoking Services are now asked to collect data on use of "unlicensed nicotine containing products" (these will primarily be e-cigarettes, and so are referred to as e-cigarettes in the questions below). If you use the North 51 'Quit Manager' database you should be able to find this information on your database, and other systems may record this too. Does your SSS have this information for **pregnant women** who report using or having used e-cigarettes *when pregnant*?

More info

- Yes
- No

5.a If **Yes**: For the year 1 April 2014 to 31 March 2015 how many **pregnant women** were recorded as using e-cigarettes?

5.a.i If **Yes**, you may also have more detailed information on e-cigarette use. If so, can you tell us how many **pregnant women** reported:

	Number of women	Information not available
Using e-cigarettes instead of licensed medication (e.g. NRT)		☐
Using e-cigarettes at the same time as licensed medication (e.g. NRT)		☐

Switching between using a licensed medication and e-cigarettes, but didn’t use them at the same time		☐

5.b If **No**:
For the year 1 April 2014 to 31 March 2015, please would you estimate the percentage of **pregnant women** who admit to using e-cigarettes

6 Does your SSS keep any additional records of e-cigarette use, e.g. particularly in pregnancy or after childbirth?

- ◯ Yes
- ◯ No

6.a If **Yes**, please briefly describe what records you keep

7 Does your SSS have a service-wide e-cigarette policy?

	Select one		
	Yes	No	If Yes, would you be willing to send us a copy of this?
In pregnancy?	◯	◯	Please select
Out of pregnancy?	◯	◯	Please select

If you responded that you are happy to send us copies of your written

policy on e-cigarettes, we would be very grateful if you could email this to *sue.cooper@nottingham.ac.uk* or send by post (address at end of survey)

8 In your SSS, how do your stop smoking practitioners generally respond to **pregnant women** who are **smoking but *not* using e-cigarettes** and **who ask whether or not it would be a good idea to use them**? (select one option)

- Very unlikely to advise using e-cigarettes
- Unlikely to advise using e-cigarettes
- Neither likely or unlikely to advise using e-cigarettes
- Likely to advise using e-cigarettes
- Very likely to advise using e-cigarettes

9 In your SSS, how do your stop smoking practitioners generally respond to **pregnant women** who **smoke and are using e-cigarettes** too? (select one option)

- Very unlikely to advise to continue using e-cigarettes
- Unlikely to advise to continue using e-cigarettes
- Neither likely or unlikely to advise to continue using e-cigarettes
- Likely to advise to continue using e-cigarettes
- Very likely to advise to continue using e-cigarettes

10 In your SSS, how do your stop smoking practitioners generally respond to **pregnant women** who have ***stopped* smoking and are using e-cigarettes**? (select one option)

- Very unlikely to advise to continue using e-cigarettes

- Unlikely to advise to continue using e-cigarettes
- Neither likely or unlikely to advise to continue using e-cigarettes
- Likely to advise to continue using e-cigarettes
- Very likely to advise to continue using e-cigarettes

Page 5: Section C: Other treatments for smoking cessation in pregnancy

This section asks for information about the kinds of support provided by your SSS between 1 April 2014 and 31 March 2015

11 We are interested in the locations used to deliver support and also the methods employed. Please indicate the approximate percentage of **pregnant smokers** who were delivered support in the ways indicated, as a proportion of all women who received support during this period.

	Support used (select all that apply)	% of women using support type	Is this value estimated? Yes	Is this value estimated? No
One-to-one support in women's homes	☐		○	○
One-to-one support in a clinic setting	☐		○	○
Telephone support	☐		○	○
Text message support	☐		○	○
Email support	☐		○	○
Other (specify below)	☐		○	○

11.a If other, please specify

In the next 2 questions (Q12 & Q13), the following definitions apply:

Single therapy NRT = prescribed only one NRT product at a time

Dual therapy NRT = prescribed NRT patch PLUS a short-acting NRT product (e.g. gum, nasal spray, lozenge)

12 Please can you tell us the percentage of **pregnant smokers** supported by your SSS who were offered each of the following interventions? **Tick boxes to indicate the types of support offered by your SSS, and select all that apply. For each ticked box, please estimate or state the proportion of women offered intervention.** You may need to estimate percentages based on your impressions; please indicate if this is estimated or not estimated, as appropriate. Some responses may be zero if treatments are not offered by your service.

More info

			Is this value estimated?	
	Support used (select all that apply)	% of women using support type	Yes	No
Behavioural support AND single therapy NRT	☐		○	○
Behavioural support AND dual therapy NRT	☐		○	○
Single therapy NRT ONLY (no expectation that women attend for other support)	☐		○	○
Dual therapy NRT ONLY (no expectation that women attend for other support)	☐		○	○
Behavioural support ONLY	☐		○	○

Other treatment or treatment combination (specify below)	☐	[ ]	○	○

12.a If Other, please specify

[ ]

13 If NRT is used, how is this first offered to **pregnant smokers** (i.e. at the start of support from your SSS)? (select one option)

More info

- ○ Single therapy
- ○ Dual therapy
- ○ Dual and/or single therapy depending on client preferences
- ○ Do not offer NRT in pregnancy

14 We are interested to know how long pregnant smokers tend to use NRT when trying to stop smoking.
Below are a list of time periods describing the length of time that they might use NRT.
Using your experience, for women who accept NRT in your SSS, please estimate and **rank the length of time that they are likely use NRT**, where **1** indicates the **most common** period of time that women use NRT for, and **5** indicates the **least common** period of time that women use NRT for.

Please don't select more than 1 answer(s) per row.

Please don't select more than 1 answer(s) in any single column.

	1 (most common period of time that women use NRT for)	2	3	4	5 (least common period of time that women use NRT for)
less than 2 weeks	☐	☐	☐	☐	☐
at least 2 weeks but less than 4 weeks	☐	☐	☐	☐	☐
at least 4 weeks but less than 6 weeks	☐	☐	☐	☐	☐
at least 6 weeks but less than 8 weeks	☐	☐	☐	☐	☐
at least 8 weeks	☐	☐	☐	☐	☐

15 Outside of pregnancy, NRT is sometimes used to help people to cut down before quitting. What best describes your SSS's approach to recommending this for **pregnant smokers**? (select one option)

- ○ Very unlikely to advise using NRT to cut down to quit
- ○ Unlikely to advise using NRT to cut down to quit
- ○ Neither likely or unlikely to advise using NRT to cut down to quit
- ○ Likely to advise using NRT to cut down to quit
- ○ Very likely to advise using NRT to cut down to quit

16 Do you have a manual or booklet which is used by your advisors to guide their delivery of **behavioural support** to **pregnant smokers**?

- Yes
- No

16.a If yes, would you be willing to send us a copy of this?

- Yes
- No

If you are happy to send us a copy of your behavioural support manual, we would be very grateful if you could email this to *sue.cooper@nottingham.ac.uk* or send by post (address at end of survey)

Page 6: Section D: Relapse after childbirth

This final section deals with the period immediately after childbirth when many pregnant women might re-start smoking. We are interested to know whether your SSS provides support to women at this time.

17 After childbirth, does your SSS routinely make contact with women who are believed to have stopped smoking in pregnancy?

- Yes
- No

18 Do SSS practitioners in your service routinely advise pregnant women on avoiding relapse after childbirth?

- Yes
- No

19 For women who stopped smoking in pregnancy, do your SSS practitioners recommend or suggest any of the following treatments to help prevent relapse **after** childbirth? (select all that apply)

- Single therapy NRT (patch)
- Single therapy NRT (short-acting)
- Dual therapy NRT (patch + short-acting)
- Varenicline
- Bupropion
- Behavioural support
- Self-help support

- [ ] E-cigarettes for relapse prevention
- [ ] Other

19.a If you selected Other, please specify:

20 What is your SSS view on using e-cigarettes to prevent relapse **after** childbirth? (select one option)

- ○ Very unlikely to advise using e-cigarettes
- ○ Unlikely to advise using e-cigarettes
- ○ Neither likely or unlikely to advise using e-cigarettes
- ○ Likely to advise using e-cigarettes
- ○ Very likely to advise using e-cigarettes

Page 7: Finally

21 If you have any additional comments or clarifications about any of the items in this survey, please mention them here.

22 **Finally – we are interested in hearing more about the views of SSS managers on the use of e-cigarettes in pregnancy. Would you be willing to be interviewed (by phone) to let us hear more about what you think on this important issue?**

- Yes
- No

22.a If **yes**, please provide contact details (name and email or phone number):

Page 8: Thank you

Thank you for taking the time to complete this survey.

If you responded above that you are happy to send us copies of your written policies on e-cigarettes and/or behavioural intervention manuals, we would be very grateful if you could email these to *sue.cooper@nottingham.ac.uk* or send hard copies to:

SSSP Survey
Smoking & Pregnancy Research Group
Division of Primary Care
University of Nottingham
Room 1406, Tower Building
University Park
Nottingham
NG7 2RD

Key for selection options

7.1.b - If Yes, would you be willing to send us a copy of this?
Yes
No

7.2.b - If Yes, would you be willing to send us a copy of this?
Yes
No
